# Double-dose icotinib may induce the emergence of the EGFR exon 20 T790M mutation in non-small cell lung cancer patients harboring EGFR-sensitive mutation

**DOI:** 10.3389/fonc.2022.898586

**Published:** 2022-07-26

**Authors:** Jianxin Chen, Xilin Wu, Junhui Wang

**Affiliations:** Department of Medical Oncology, The Quzhou Affiliated Hospital of Wenzhou Medical University, Quzhou People′s Hospital, Quzhou, China

**Keywords:** icotinib, double dose, T790M mutation, lung cancer, epidermal growth factor receptor

## Abstract

**Background:**

Acquired resistance to epidermal growth factor receptor tyrosine kinase inhibitors (EGFR-TKIs) inevitably occurs in non-small cell lung cancer (NSCLC) patients harboring EGFR-sensitive mutations. There are approximately half of the patients who developed resistance to EGFR-TKIs treatment, the mechanism of which remains undiscovered. We occasionally found that double-dose icotinib as further-line salvage treatment may induce the emerging mutation of EGFR exon 20 T790M in NSCLC patients. The present study, therefore, was conducted to explore the probability of the emerging T790M mutation after exposure to double-dose icotinib in metastatic NSCLC patients.

**Patients and Methods:**

Metastatic NSCLC patients who received double-dose icotinib as salvage treatment after progression on first-generation TKIs and systematic chemotherapy were screened. Thereafter, patients who received a repeated next-generation sequencing (NGS) test with tumor sample were further enrolled. The procedure of NGS was performed with the standard criteria. Finally, the clinical characteristics, treatment procedures, and outcomes of eligible patients were reviewed and presented.

**Results:**

Three patients have been detected with the emerging T790M mutation after double-dose icotinib exposure, with a mutation frequency of 19.6%, 8.2%, and 87.5%. During the treatment of targetable TKIs including almonertinib or osimertinib, partial response was observed in two patients, and stable disease was observed in the other. The progression-free survival by targetable TKIs for the patients was 3.7+ months (still in extension), 4.9+ months (still in extension), and 6.3 months. Manageable adverse events were observed during the treatment of TKIs.

**Conclusion:**

The results of the present study revealed that the emerging EGFR exon 20 T790M mutation might be induced by double-dose icotinib exposure in further-line treatment. Patients with the emerging T790M mutation responded well to the treatment of targetable TKIs including almonertinib or osimertinib.

## Introduction

Epidermal growth factor receptor tyrosine kinase inhibitors (EGFR-TKIs) are the recommended treatment strategy in patients with non-small cell lung cancer (NSCLC) harboring EGFR-sensitive mutations, especially with common mutations including exon 19 deletion and exon 21 L858R mutation (1–4). However, acquired resistance to EGFR-TKIs inevitably occurs after 10 to 14 months of exposure. The most common mechanism of acquired resistance was the EGFR p.Thr790Met point mutation (T790M), which accounts for 40% to 50% (5, 6). Among patients with the acquired EGFR T790M mutation, osimertinib, an irreversible EGFR-TKI selective for both EGFR-sensitive mutations and T790M resistance mutations, has emerged as a standard treatment choice, with an objective response rate (ORR) of 61% and a progression-free survival (PFS) time of 12.3 months (7, 8). However, there are still approximately 50% of patients who developed resistance to EGFR-TKIs treatment, the mechanism of which remains unknown (9). Although systematic therapy with cytotoxic agents has been suggested as standard therapy for those patients without the T790M mutation, confined efficacy accompanied with related adverse events significantly limited its application, especially for patients with poor performance status.

Icotinib, a highly specific and selective EGFR-TKI, has been approved for treatment of advanced or metastatic NSCLC patients harboring EGFR-sensitive mutations in China (10). Recently, a randomized phase II trial (INCREASE), conducted to evaluate the efficacy and safety of double-dose icotinib as front-line treatment in NSCLC patients harboring EGFR exon 21 L858R mutation, reported its positive results (11). The results of the study demonstrated tolerable toxicities, as well as better PFS [12.9 versus 9.2 months, HR, 0.75; 95% confidence interval (CI): 0.53–1.05, *p* < 0.05] of double-dose icotinib in NSCLC patients with the EGFR exon 21 L858R mutation when compared with routine dose (11). However in the INCREASE trial, the probability of the acquired T790M mutation, as well as the subsequent treatment strategies between double-dose and routine-dose icotinib, was not reported. In clinical practice, we occasionally found that double-dose icotinib as further-line salvage treatment may induce the emerging/increasing mutation of EGFR exon 20 T790M in NSCLC patients (12). To address the question, we therefore performed a retrospective study to explore the probability of the emerging T790M mutation after exposure to double-dose icotinib in previous T790M-negative NSCLC patients.

## Materials and methods

### Patients

Patients were eligible to enroll in the present retrospective study if they met the listed criteria: (1) patients diagnosed with metastatic NSCLC harboring EGFR-sensitive mutations (exon 19 deletion/exon 21 L858R mutation) between July 2018 and December 2021 in our institute; (2) after progression on first-line treatment with first-generation EGFR-TKIs (without the acquired T790M mutation/or T790M mutation by frequency less than 5%) and systematic chemotherapy, patients who received double-dose icotinib as salvage treatment were further selected; (3) in addition, patients who received a repeated NGS test with tumor sample after double-dose icotinib exposure were finally enrolled; and (4) efficacy and safety of the treatment with third-generation TKIs should be evaluated by RECIST 1.1 and NCI CTCAE 4.0. The clinical and molecular characteristics including sex, age, Eastern Cooperative Oncology Group performance status (ECOG PS), disease stage, smoking history, brain metastases, EGFR mutations, and concurring mutation were reviewed. EGFR mutation testing was performed using commercially available next-generation sequencing (NGS) analysis (BioMed Diagnostics, China). The NGS tests (before/after double-dose icotinib) were performed using tumor tissue obtained from same-site biopsy to reduce the incidence of false-negative results by plasma or tumor heterogeneity by tumor location. Available gene tests in the NGS panel adopted in the present study included anaplastic lymphoma kinase (ALK), V-Ki-ras2 Kirsten rat sarcoma viral oncogene homolog (KRAS), ROS proto-oncogene 1 (ROS1), neuroblastoma RAS viral oncogene homolog (NRAS), V-raf murine sarcoma viral oncogene homologB1 (BRAF), RET proto-oncogene (RET), receptor tyrosine-protein kinase erbB-2 (ERBB2), RAC-alpha serine/threonine-protein kinase (AKT1), MNNG HOS transforming gene (MET), discoidin domain receptor tyrosine kinase 2 (DDR2), phosphatidylinosito-4,5-bisphosphate 3-kinase (PIK3CA), fibroblast growth factor receptor 1 (FGFR1), phosphatase and tensin homolog (PTEN), and mitogen-activated protein kinase 1 (MAP2K1). The present study was approved by the Ethical Committee of People′s Hospital of Quzhou. All experiments in the present study were performed in accordance with international ethical guidelines.

### Next-generation sequencing analysis

For NGS analysis, the library was performed based on the OncoAim™ cancer 15 gene panel (BioMed Diagnostics, Inc., Shanghai, China). DNA fragment shearing was developed with Covaris M220, followed by phosphorylation, and adaptor ligation. The quality of the fragments was evaluated with bio-analyzer high-sensitivity DNA assay. The indexed samples were sequenced on Hiseq500 sequencer (Illumina, Inc., USA) with paired reads of 150 bp length. Local alignment optimization, variant calling, and annotation were conducted with GATK 3.2, MuTect, and VarScan; Tophat 2 and Factera 1.4.3 were adopted for the DNA translocation analysis.

## Results

**Figure 1:**
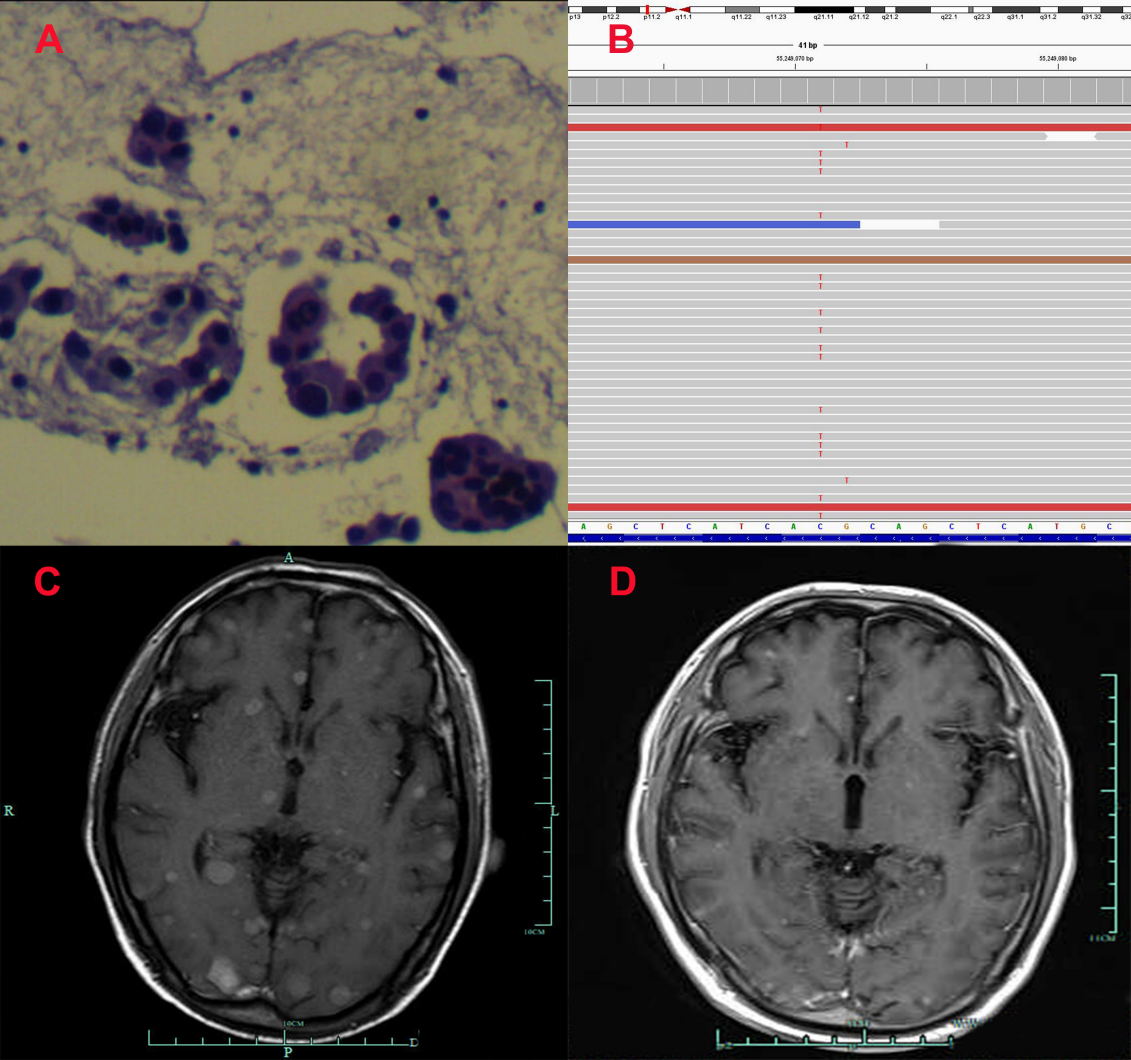
Pathological and clinical findings of Patient 1. **(A)** Pathological findings with HE staining. **(B)** NGS screenshots suggested emerging EGFR exon 20 T790M mutations. **(C)** Brain MRI image before salvage almonertinib treatment (after the emerging T790M mutation). **(D)** Brain MRI image after salvage almonertinib treatment.

**Figure 2:**
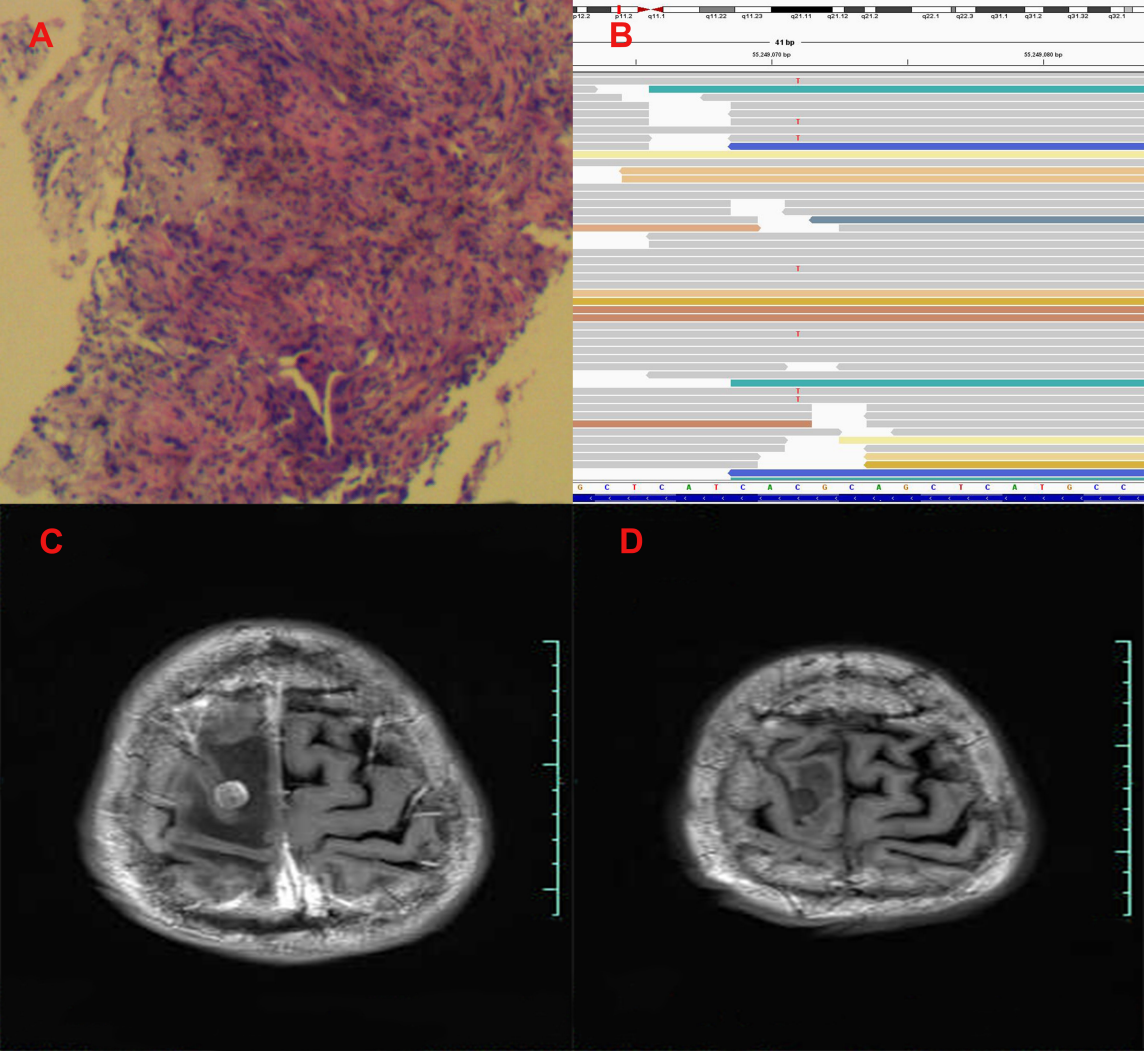
Pathological and clinical findings of Patient 2. **(A)** Pathological findings with HE staining. **(B)** NGS screenshots suggested emerging EGFR exon 20 T790M mutations. **(C)** Brain MRI image before salvage almonertinib treatment (after the emerging T790M mutation). **(D)** Brain MRI image after salvage almonertinib treatment.

**Figure 3:**
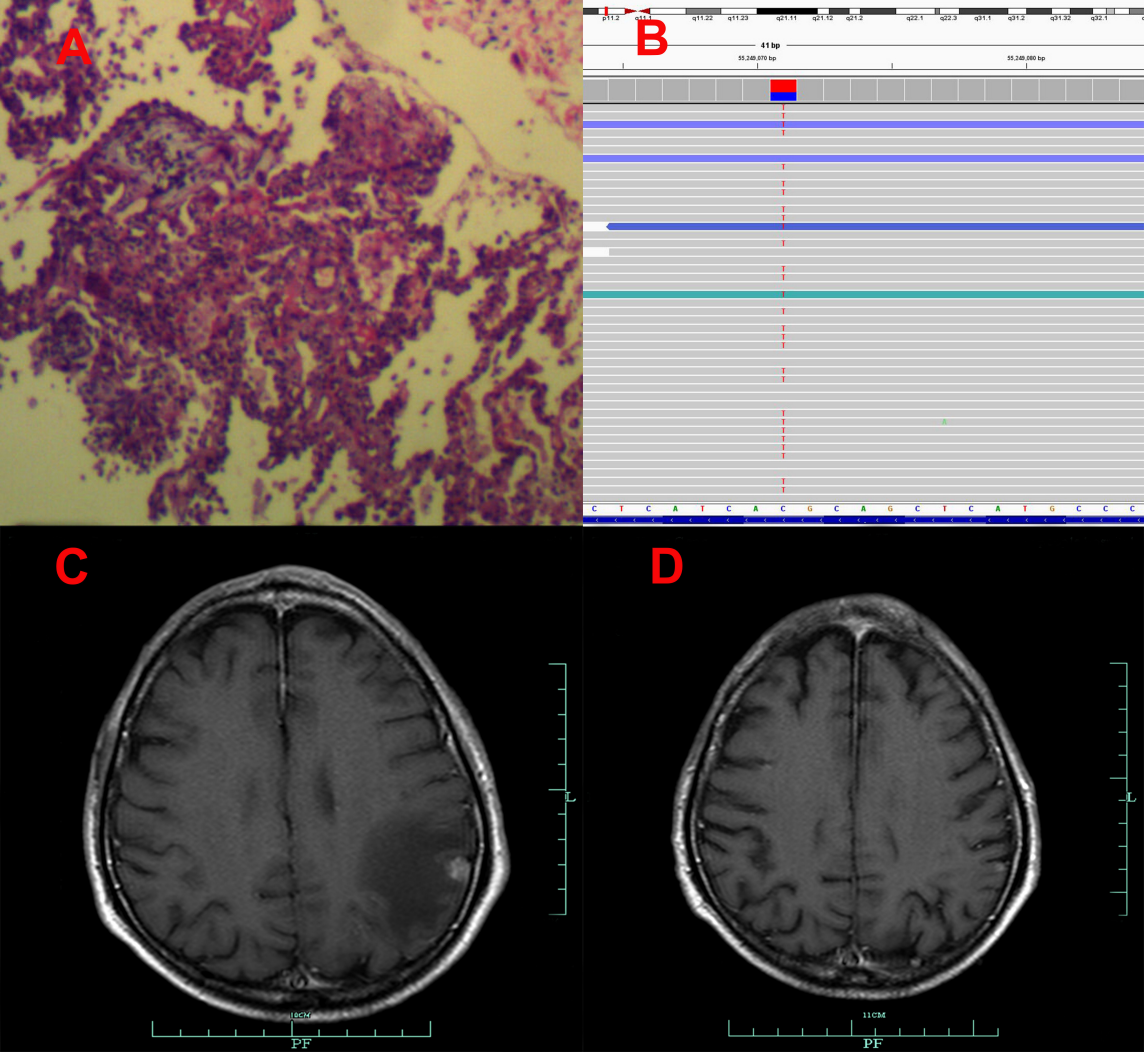
Pathological and clinical findings of Patient 3. **(A)** Pathological findings with HE staining. **(B)** NGS screenshots suggested increasing EGFR exon 20 T790M mutations (4% up to 87.5%). **(C)** Brain MRI image before salvage almonertinib treatment (after the emerging T790M mutation). **(D)** Brain MRI image after salvage osimertinib treatment (12).

Between July 2018 and December 2021, 162 patients with metastatic NSCLC harboring EGFR-sensitive mutations (exon 19 deletion/exon 21 L858R mutation) were screened with electronic medical record system. After first-line administration of EGFR TKIs and systematic chemotherapy (with or without antiangiogenic agents), there were 11 patients who received double-dose icotinib as salvage treatment without the EGFR exon 20 T790M mutation. However, there were only four patients who received repeated NGS analysis after progression on double-dose icotinib. Finally, three patients were detected with the emerging/increasing T790M mutation after double-dose icotinib exposure (**Figures 1A**, **2A**, and **3A**). Clinical and molecular characteristics including sex, age, ECOG PS, disease stage, smoking history, brain metastases, EGFR mutations, and concurring mutation are summarized in **Table 1**.

**Table 1:** Baseline demographic and clinical/molecular characteristics of patients.

	Patient 1	Patient 2	Patient 3
Sex	Male	Male	Male
Age	59	62	67
ECOG PS	2	1	2
Stage	IVB	IVB	IVB
Smoking history	No	Yes	Yes
Brain metastases	Yes	Yes	Yes
EGFR mutation	Exon 21 L858R	Exon 19 deletion	Exon 19 deletion
Concurring mutation	TP53 S240G	ND	ND

ECOG PS, Eastern Cooperative Oncology Group Performance Status; EGFR, Epidermal Growth Factor Receptor; ND, Not Detected.

**Figure 4:**
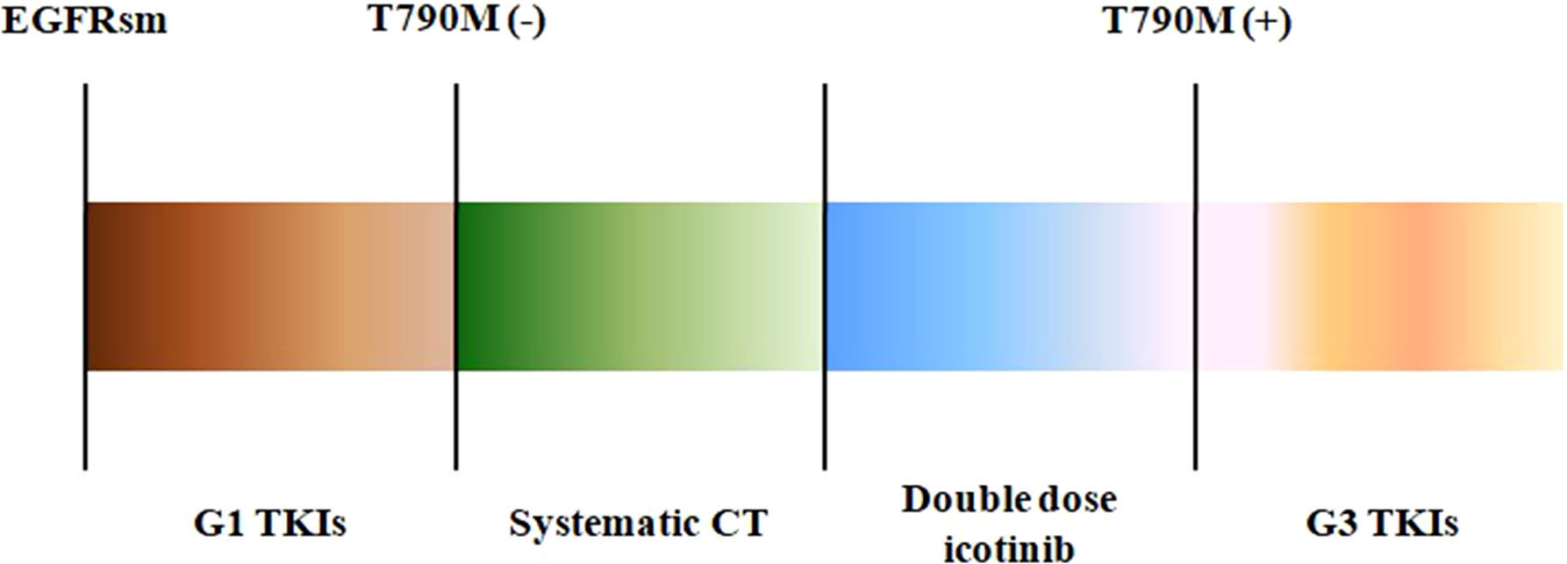
The treatment procedures of the three patients. EGFRsm, epidermal growth factor receptor sensitive mutation; TKIs, tyrosine kinase inhibitors; G1, first-generation TKIs; G3, third-generation TKIs; CT, chemotherapy.

Among the three included patients, two patients (Patient 1 and Patient 2) received gefitinib as first-line treatment, and the other one (Patient 3) received routine-dose icotinib as first-line therapy. The progression-free survival (PFS) time of the first-line TKIs for the three patients were 14.3 months, 21.6 months, and 8.1 months. The acquired T790M mutation was not detected by repeated NGS analysis using tumor tissue in two patients (Patient 1 and Patient 2). The other patient (Patient 3) was detected with the T790M mutation with a low frequency of 4.0%. Thereafter, all patients received systematic chemotherapy (Patient 3 received osimertinib followed by chemotherapy) as subsequent treatment. After progression, a further NGS test using fresh tumor specimen by re-biopsy was developed in two patients (Patient 1 and Patient 2), the results of which failed to detect the EGFR exon 20 T790M mutation. As salvage treatment, all patients received double-dose icotinib administration. As a result, two patients (Patient 1 and Patient 2) were evaluated to have disease progression on double-dose icotinib treatment in the first examination (PFS: 2.7 months and 1.6 months, respectively). Disease progression in brain was discovered in all of the three patients (**Figures 1C**, **2C**, and **3C**). A repeated NGS analysis was conducted again, the results of which suggested the emerging/increasing T790M mutation in the three patients, with a frequency of 19.6%, 8.2%, and 87.5% (**Figures 1B**, **2B**, and **3B**), respectively. Based on that, third-generation TKIs, including almonertinib and osimertinib, were prescribed as further-line treatment. Consequently, partial response was observed in two patients (Patient 1 and Patient 2), and stable disease was observed in Patient 3 (**Figures 1D**, **2D**, and **3D**). The PFS time by third-generation TKIs for the patients was 3.7+ months (still in extension), 4.9+ months (still in extension), and 6.3 months by 30 November 2021. In addition, an adverse event, cerebral infarction, was observed in Patient 2. Procedures, efficacy, and safety assessment of treatment for the three patients are presented in **Table 2**. The pathologic diagnosis, emerging T790M mutations, and efficacy evaluation in brain for the three patients are presented in **Figures 1**–**3**, respectively. The treatment procedure of the patients is presented in **Figure 4**.

**Table 2:** Procedure, efficacy, and safety assessment of treatment for the patients.

	Patient 1	Patient 2	Patient 3
First-line TKIs	Gefitinib	Gefitinib	Icotinib
PFS for first-line TKI	14.3 months	21.6 months	8.1 months
PD lesions for TKI	Lung/pleura	Lung/lymph node	Lung/lymph node
PFS for double-dose icotinib	2.7 months	1.6 months	6.2 months
AEs for double-dose icotinib	Ileus	No	No
Frequency for the emerging T790M	19.6%	8.2%	87.5%
Salvage TKIs for T790M	Almonertinib	Almonertinib	Osimertinib
Best response for the salvage TKIs	PR	PR	SD
AEs for the salvage TKIs	No	Cerebral infarction	No
PFS for the salvage TKIs	3.7+ months	4.9+ months	6.3 months

TKI, tyrosine kinase inhibitor; PFS, progression-free survival; PD, progressive disease; AE, adverse event; T790M, EGFR exon20 T790M mutation; PR, partial response; SD, stable disease. +, the survival time is still in extension.

## Discussion

In the current study, our results indicated that there were selected NSCLC patients without the acquired EGFR exon 20 T790M mutation, but who might be induced by double-dose icotinib exposure in further-line treatment. Patients with the emerging T790M mutation responded well to third-generation TKIs including almonertinib or osimertinib.

In recent years, osimertinib has been recommended as the preferred first-line EGFR TKIs in metastatic NSCLC patients with EGFR-sensitive mutations because of the positive results of the trial FLAURA (13). However, in clinical practice, there were still several questions to be addressed. Firstly, there was a slight statistical difference on OS benefits for Asian patients receiving osimertinib as first-line treatment compared to gefitinib according to subgroup analysis (HR, 1.0; 95% CI, 0.75–1.32) (13). In the further expanded research for the Chinese cohort, there was still no statistical difference observed (HR, 0.85; 95% CI, 0.56–1.29) (14). Secondly, after progression on first-generation TKIs, there were approximately 50% patients who may develop the acquired T790M mutation, who could obtain another opportunity to receive further-line osimertinib treatment, resulting in a PFS of 10.1 months according to the AURA 3 study (15). However, in patients who receive osimertinib as front-line treatment, systematic chemotherapy might be the only standard choice after progression, which usually resulted in limited efficacy but significant adverse events. Finally, the first-line choice of osimertinib might be more expensive than gefitinib/icotinib followed by osimertinib. The cost of medication is a significant aspect for making a final choice, especially in developing countries including China. Hence, in real-world practice, there are still a majority of patients receiving the regimen of gefitinib/icotinib followed by osimertinib. In such a situation, acquired resistance to first-/second-generation EGFR TKIs inevitably occurs, with molecular mechanisms remaining challenging. Currently, acquired resistance mechanisms to EGFR TKIs can be classified into EGFR-dependent mechanisms and EGFR-independent mechanisms, in which the EGFR exon 20 T790M mutation accounts for approximately 40% to 50% (16). Patients with the EGFR exon 20 T790M mutation responded well to third-generation TKIs including almonertinib and osimertinib, which is said to be a “lucky mutation”. However, one-half of the patients still had a T790M-negative mutation; they commonly received chemotherapy, usually resulting in limited efficacy. Immune checkpoint inhibitors (ICIs) and anti-PD-1/PD-L1 monoclonal antibodies inhibit the interaction of PD-1 with their ligands, hence promoting an immunologic response by T cells against cancer cells, which failed to prove its efficacy as mono-therapy in patients harboring EGFR-sensitive mutations after resistance (17). Prospective clinical trials of combinational therapy of cytotoxic drugs combined with ICIs, including KEYNOTE 789 and CHECKMATE 722, are still ongoing. Current treatment strategies for NSCLC patients with T790M mutation-free TKIs resistance have not met clinical demands.

Recently, there was a prospective phase II clinical trial conducted to investigate the efficacy and safety of gefitinib as further-line re-challenge treatment for advanced NSCLC patients with EGFR-sensitive mutations (18). The study included stage IIIB/IV NSCLC patients with EGFR common mutations including exon 19 deletion and exon 21 L858R mutation, who had benefited from first-line gefitinib treatment, and followed by further-line chemotherapy. Eligible patients received routine-dose gefinitib as re-challenge treatment. The results of the research showed that predefined disease control rate (DCR) was achieved in 69.8% (95% CI, 49.87–74.91) of patients. However, objective response rate (ORR) was reported merely in 4.7% (95% CI, 0.78–13.06) of patients, which suggested that anti-tumor efficacy of re-challenge of gefitinib was not satisfactory among the majority of the patients (18). In the present study, we also found that 2/3 patients (Patient 1 and Patient 2) suffered rapid progression of disease during the exposure of double-dose icotinib, the results of which were consistent with the former publication (18). However, we detected emerging T790M mutations unexpectedly after the exposure to double-dose icotinib, which was rarely reported in former literature. It should be pointed out that the NGS test using fresh tumor tissue from re-biopsy was also performed before the prescription of double-dose icotinib, the results of which did not reveal the T790M mutation. Based on this, it was most likely that the emerging T790M mutation was induced by the administration of double-dose icotinib. With the detection of the emerging T790M mutation, third-generation TKIs were prescribed as salvage treatment, which resulted in significant efficacy among the three patients. Therefore, the emerging T790M mutation might also be considered as a predictor to targetable TKIs. In addition, the emerging T790M mutations after the re-challenge of gefitinib in the CTONG 1304 trial were also reported (18). The authors reported that T790M-positive patients increased significantly (from 11 to 23, *p* = 0.0081) after the gefinitib re-challenge treatment. However, the sample adopted for the NGS test in that study was plasma, which may lead to a false-negative result before the re-challenge treatment of gefitinib. In the present study, fresh tumor tissue from re-biopsy was used for the NGS analysis, which may decrease the possibility of a potential false-negative result. Even so, we still suggested that a prospective, well-designed clinical trial might be essential to further address the issue of the emerging T790M mutation after double-dose icotinib treatment. In fact, the aforementioned study has been designed, with eligible patients being recruited in our institute.

Although the mechanism of the emerging T790M mutation after double-dose icotinib exposure remained unknown, we supposed that tumor heterogeneity might be the probable reason. In the AURA trial, 21% of patients with the T790M-negative mutation still responded to osimertinib, the specific T790M mutation inhibitor, which suggested that the T790M-negative results might appear to be the false-negative ones. The extremely low frequency of the T790M mutation led to false results (7). In addition, the TREM trial was conducted to evaluate the efficacy of osimertinib in patients progressing on standard EGFR-TKI treatment regardless of T790M status, the results of which showed that ORR was 28% (15% to 41%) for T790M-negative patients, with a PFS of 5.1 months (19). Hence, it was suggested that the T790M mutation could not be detected in a portion of patients, which may still respond to targetable TKIs. In the present study, the double-dose exposure of icotinib may rapidly eliminate T790M-negative subpopulations, which resulted in the elevated portion of the T790M mutation. For all these, basic experiments may still be essential to investigate the potential mechanisms of the emerging T790M mutations.

In addition, gefitinib and icotinib were approved as first-line treatment in metastatic NSCLC patients harboring EGFR-sensitive mutations because of their phase III, randomized research of the IPASS trial and the CONVINCE trial, respectively (4, 10). However in the present study, patients received gefitinib as first-line treatment, whose PFS was much better than that of icotinib. Based on the limited sample size, we still suggested that the difference in PFS between gefitinib and icotinib was coincidental. A “head to head” research (ICOGEN) was conducted to compare the efficacy between icotinib and gefitinib, the results of which did not suggest a statistical difference between icotinib and gefitinib (HR, 0.835; 95% CI, 0.667–1.046) (20). The comparative results among the first-line TKIs should be prudently concluded. Moreover, icotinib rather than other TKIs was adopted in the present study to induce treatment for emerging T790M mutations. In clinical practice, we have observed that the high-dose exposure of first-generation TKIs might be easier to induce that. Icotinib was the only TKI whose double-dose administration has been approved based on its clinical trial (11). However, for the other TKIs including gefitinib, erlotinib, and afatinib, high-dose exposure was not approved, which might lead to potential extra toxicities. This was the main reason why icotinib was chosen to induce treatment in the present study.

There were several limitations in the present study. The small sample size is the most obvious one. Owing to the frequent NGS tests using fresh tumor tissue, as well as the expensive cost of the serial gene tests, a majority of patients were excluded in the present retrospective study. We have noticed that the small sample size might limit its reliability in the present study. To address this, we have sponsored another prospective cohort study to further confirm the clinical phenomenon. In addition, the nature of a retrospective study may lead to a selection bias for the interpretation of the results.

## Conclusion

In total, the results of the present study revealed that the EGFR exon 20 T790M mutation might be induced by double-dose icotinib exposure as further-line treatment in patients with former T790M-negative mutation. Patients with emerging T790M mutations responded well to the treatment of targetable TKIs including almonertinib or osimertinib.

## Data availability statement

The original contributions presented in the study are included in the article/supplementary material. Further inquiries can be directed to the corresponding author.

## Ethics statement

The studies involving human participants were reviewed and approved by Ethical Committee of People′s Hospital of Quzhou. The patients/participants provided their written informed consent to participate in this study.

## Author contributions

JC: Conceptualization, Methodology, Software, Writing- Original draft preparation, Software, Validation. XW: Data curation, Supervision. JW: Visualization, Investigation, Writing-Reviewing and Editing. All authors contributed to the article and approved the submitted version.

## Funding

The study was supported by Instructional Project of Quzhou (2020057).

## Acknowledgments

The authors thank the patients for their participation and agreement to publish the study.

## Conflict of interest

The authors declare that the research was conducted in the absence of any commercial or financial relationships that could be construed as a potential conflict of interest.

## Publisher’s note

All claims expressed in this article are solely those of the authors and do not necessarily represent those of their affiliated organizations, or those of the publisher, the editors and the reviewers. Any product that may be evaluated in this article, or claim that may be made by its manufacturer, is not guaranteed or endorsed by the publisher.
